# Analysis of alcoholism data using support vector machines

**DOI:** 10.1186/1471-2156-6-S1-S136

**Published:** 2005-12-30

**Authors:** Robert Yu, Sanjay Shete

**Affiliations:** 1 Department of Epidemiology, Unit 1340, The University of Texas M. D. Anderson Cancer Center, Houston, TX 77030 USA

## Abstract

A supervised learning method, support vector machine, was used to analyze the microsatellite marker dataset of the Collaborative Study on the Genetics of Alcoholism Problem 1 for the Genetic Analysis Workshop 14. Twelve binary-valued phenotype variables were chosen for analyses using the markers from all autosomal chromosomes. Using various polynomial kernel functions of the support vector machine and randomly divided genome regions, we were able to observe the association of some marker sets with the chosen phenotypes and thus reduce the size of the dataset. The successful classifications established with the chosen support vector machine kernel function had high levels of correctness for each prediction, e.g., 96% in the fourfold cross-validations. However, owing to the limited sample data, we were not able to test the predictions of the classifiers in the new sample data.

## Background

Alcoholism is a complex genetic disease, and the Collaborative Study on the Genetics of Alcoholism (COGA) has been extensively investigating its underlying mechanisms. Like many other complex diseases, alcoholism presents extraordinary challenges to both the diagnostic categorization of individuals with alcoholism-related traits and the genetic analyses of these traits using conventional linkage analysis approaches because the disease does not have a simple single-locus etiology. Analyses of complex diseases involve the investigation of multiple genes and their interactions in contribution to the susceptibility to the disorder. In this study, we addressed these issues by exploring an approach to reduce the size of the marker set and computational burden in the further linkage analyses.

In light of the recent development and successful application of machine learning theories in other areas, we hypothesized that support vector machine (SVM) [1] is an ideal method to be adapted to the alcoholism dataset provided by COGA for the Genetic Analysis Workshop 14. To test this hypothesis, we focused on the microsatellite dataset for the relatively lower requirement of experimental computation resource. We believe the resultant method established on the microsatellite dataset could be adapted to large single-nucleotide polymorphism datasets.

In theory, for a given training data (*x*_*i*_,*y*_*i*_), where *x*_*i *_is the *i*^th ^record, there is a vector of attributes with *m *elements (also called *m *dimensions), and *y*_*i *_is the corresponding categorical value (usually a binary value). A binary classifier called a hyperplane – a plane in a high-dimension space only separating data dots into two subspaces – can be built up to separate the class members of the datasets. However, real-world problems involve inseparable data for which such a hyperplane does not exist [1,2]. One solution is to map the data into a higher-dimensional space (called a feature space) and define a hyperplane there. This binary classifier hyperplane can be linear if the feature space reaches a sufficiently high dimension. However, as the feature space dimension increases, both computational and learning-theoretic costs are incurred, and the learning system is exposed to the risk of finding trivial solutions, i.e., of overfitting. To overcome these difficulties, the SVM chooses the maximum margin that allows for separating the hyperplane from other choices while avoiding overfitting. SVM uses kernel functions for the data mapping between input space and feature space, which significantly decreases the computational burden. When a dataset contains mislabeled examples or an inappropriate kernel function was chosen, the SVM might have difficulty separating groups. The former problem can be addressed by using a soft margin that allows some training examples to fall on the wrong side of the separating hyperplane. Thus, two parameters are needed to adjust the SVM according to the types of datasets undergoing classification: the kernel function and the magnitude of the penalty for violating the soft margin. Besides errors in these parameters, an imbalance in the number of positive and negative training examples, in combination with noise in the data, is likely to cause the SVM to make incorrect classifications. So does the situation in which the number of attributes is far greater than the number of total records in the training data are likely to cause the SVM to make incorrect classifications.

The SVM is a supervised learning method, which has the ability to weigh input features, e.g., markers, according to their relevance to the classification scheme as determined through the learning process [1-3]. Once learning is complete, in the form of an established hyperplane, SVM could be used to generalize to the unseen data, i.e., to make a prediction.

## Materials

We chose 315 microsatellite markers on 22 autosomal chromosomes with 12 different phenotype variables. The 22 sub-datasets of each chromosome were merged into one single genome dataset and then were appended with a chosen phenotype variable for a specific session of the SVM run.

### Phenotype variables

The 12 phenotype traits (Table 1) were selected on the basis of the following considerations: the trait is convenient for the preliminary analysis; there is less missing data for the chosen variable; it is easy to organize the data according to the trait (e.g., binary form); the results can be compared with those from other analyses; and the results are representative of our analysis methods. The genotypes of the microsatellite marker data were transformed into numeric codes based on the types of the original allele pair.

### Random sub-datasets

We then randomly created two types of sub-datasets: the random block and random set. The random block was generated by randomly choosing a number, called the block size, from the spectrum of genomic markers (total 315 across all 22 autosomal chromosomes) or from a specified number of markers (in order to limit the resulting file size to accelerate the subsequent computation) and then randomly choosing a number as a starting position within the 315 spots. The resulting file will then contain a block of continuous markers from the starting spot to the block size. The random set is generated by randomly choosing a number, called the set size, between 1 and the total number of markers (in this case, 315), and then randomly choosing spots within this spectrum of genomic markers, without duplication, until the number of picked spots equals the set size. The resulting file will thus contain a subset of the 315 markers in a "sporadic" pattern across the whole genome. Each of the two types of random sub-datasets was created in a number from 5,000 to 20,000 for each of the 12 single-phenotype-assigned datasets.

### Sequential "walk" of sub-datasets

For systematically screening the continuous block of markers, we designed another method of generating sub-datasets by determining a block size and then sequentially "walking" through from the first marker to the last. The block size could be 1 to 315 in order to cover the entire genome. However, owing to the huge size of the resulting data files and the great demand of this approach on the computing resources, we were able to perform only a limited analysis.

### SVM analysis

For this study, we focused on the dot kernel, in the form of *K*(*x*,*y*) = x * *y*, and the polynomial kernels as *K*(*x*,*y*) = (x * *y *+ 1)^d^, where *d *is the degree that defines *d*-fold interactions between attributes, i.e., . In our preliminary studies, we examined the dot kernel and the polynomial kernels using various degrees for a total of 315 microsatellite markers on 22 autosomal chromosomes with 12 different phenotype variables. Increasing the degree of the kernel function improves the separation power of the SVM; however, a higher-degree kernel function will reach a stage where the underlying dataset becomes inseparable owing to an over-learning problem. Thus, a bound on kernel function choice and distinction in data separability can be established.

### SVM program

The SVM program that we used was MYSVM, version 2.1 for Windows and version 2.1.4 for UNIX/Linux, which was written by Rüping [4] and downloaded from . All data were analyzed using four-fold cross-validations and kernel functions of dot and polynomials with degrees ranging from 1 to 6 (in some cases, degrees of polynomial kernels went to 7 through 9, 12, and 20). In the four-fold cross-validation of each sub-dataset, data were randomly partitioned into four roughly equal parts and underwent SVM analysis four times. In each analysis, one-quarter of the data was left out for prediction based on the learning/training of the remaining three-quarters of the data. A positive result was achieved when each of the four cross-validations yielded a pre-determined percentage of correct predictions, e.g., 99%. The marker sets were then used to identify the corresponding genome region.

### Missing values

The records were deleted if either the appended phenotype variable was missing or the record itself contained more than a certain percentage, e.g., 20%, of missing values among all attributes (markers). The remaining missing values were represented using -9, -999, or -1 (we have not yet experimented with them meaningfully to determine the effect of different values on the SVM analysis results). As a result, the maximum number of individuals (records) was 1,204 (versus 1,614 in the original data) and the minimum was 984 (Table 2).

## Results

As we expected, the analysis of data for the deceased phenotype variable yielded null results in both random block and random set sub-datasets. Most outputs from MYSVM showed zero members in trait class assigned value 1 during data partition for the four-fold validation. This was because there were only two members in this group. This verifies the fact that imbalanced data are difficult for SVM to separate.

**Figure 1:**
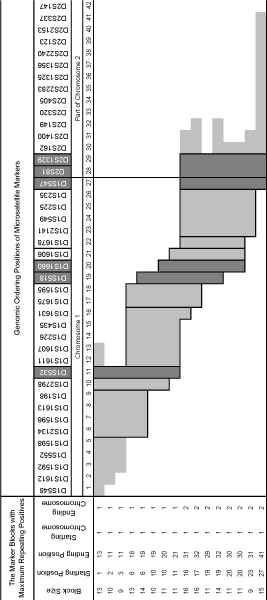
Results of microsatellite markers pattern/association with ALDX with maximum (4) repeating positives from SVM analysis using 4 different kernels.

On the other hand, our analytical runs of MYSVM for the other phenotype variables all generated various percentages of positive results. From a series of randomly generated random block sub-datasets with ALDX phenotypes, for example, we performed MYSVM analyses with polynomial kernel functions of various degrees (from two through seven). About 43% of the output files showed positive results with some degree of the polynomial functions when the percentage of correctness was set at greater than 96%. However, about 38% of them showed repeated positive results when running three different polynomial kernel functions, and about 16% showed repeated positive results with four or more different kernels (the maximum in this run of the analysis). Figure 1 shows a selected collection of the results from one of the groups picked. From a vast number of groups (which are not presented here), we observed that the positive results were only from block sizes of six or higher. (Our partial run of sequential walks using block sizes of one through four failed to yield positive results at trials with several different kernel functions, which confirms this observation.) In the results presented here, the smallest block size was nine. We also observed that the blocks that show positive results appeared more frequently in the regions of chromosomes 1, 2, and 6.

In Figure 1, the blocks and their covering marker regions are shown in light gray and the more frequently covered regions are framed. The markers with the heaviest coverage are dark gray with frames. These regions and markers are considered to show association with the ALDX phenotype trait using this categorization (i.e., 1, 2, and 3 as class 1, and 5 as class 2). When briefly comparing these association markers with those reported from the linkage analyses from other studies, we find some are matching with the linkage result, e.g., D1S532, which shows a high LOD score in a previous analysis [5].

Similar results are found not only in the remaining regions of the genome for this ALDX phenotype trait but also in the whole genome for other phenotype variables. We believe SVM is effective in recognizing the marker pattern and predicting the phenotypic traits, thus making it possible to reduce the size of the dataset.

## Discussion

In this study, we used SVM methodology as an initial exploration technique for alcoholism data. There are many details both in study design and data processing that need to be adjusted. However, our study shows that this approach is useful for association studies and for detecting causal genes in future efforts.

The COGA microsatellite dataset has enough markers and samples to allow us to test different search strategies for SVM analysis. For example, the SVM is sensitive to the specific composition of the dataset that was used in the analysis, i.e., the number of markers and the number of individual records. Changing one marker or record in a dataset successfully separated by SVM could result in changes in the next SVM analysis. Exhausting (full coverage of) the entire marker set (feature space) during the SVM analyses is still a challenge and needs further exploration. Genetic algorithms have been shown to achieve better coverage in searching the feature space. However, developing a proper fit function will be the key, and this is, as yet, case dependent. Otherwise, SVM produces the search that leads to local optimums. Nonetheless, we consider this direction worth investigating to make the SVM method more suitable to the true large dataset.

The resulting subsets of markers in each successful SVM analysis from the dataset contain both "hot spots," i.e., markers with high LOD scores in other linkage analyses, and no regions of linked markers. But we have not developed or yet seen in the reports of other data mining studies a testing method for proving the enclosure of the "hot spots" after reduction by such a data mining method as SVM. At this stage, we would suggest that the reduced marker sets in these SVM-separated datasets at least provide the hypothesis that some of the markers in the resulting set are possibly associated with the corresponding phenotypic trait, which can be tested in the replication datasets. A focus of linkage or association analysis starting with these reduced datasets would be beneficial in cases in which computation resources are heavily restricted and would reduce the number of tests significantly. SVM also proves its efficiency and high capability in searching and analyzing data genome-wide. Such an advantage is even more valuable when analyzing multi-gene or gene × gene interaction diseases such as alcoholism.

In this study, we did not have the opportunity to develop the SVM method to make full use of the information contained in the provided dataset. Although we noticed that similar situations exist in other data mining studies, we believe that to integrate information such as that derived from the pedigree and the IBD sharing values into the input features of the SVM will greatly enhance the final results. Such pre-analysis data preparation, including the proper selection of a coding system for the input genotype data, will improve the resolution of SVM analyses.

## Conclusion

The SVM is an effective approach for association analysis, data reduction, and pattern recognition when a given dataset is large. This is especially advantageous as technology has driven the information yield to a higher level and we are facing more and more overgrown-sized datasets. On the other hand, SVM has not yet been fully explored in genetic studies. Tailoring data preparation and information transformation in a way suitable to SVM analysis still remains to be done.

## Abbreviations

COGA: Collaborative Study on the Genetics of Alcoholism

SVM: Support vector machine

## Authors' contributions

RY conceived the study, participated in the computing and analysis, and drafted the manuscript. SS supervised the study, contributed to the intellectual content and analysis and revised the manuscript.

## Figures and Tables

**Table 1:** Twelve phenotype variables

No.	Label name of phenotypic traits	Description
1	Deceased	Individuals who are deceased
2	ALDX	A combination of ALDX1 and ALDX2
3	Binge	Ever binge drink
4	Blackouts	Blackouts (3 or more)
5	Morning	Morning drinking
6	Craving	Craving
7	Pers	Persistent desire to stop drinking
8	Narrow	Narrowing of drinking repertoire
9	GUATD	Stands for "give up activities to drink"
10	WDSX	Stands for "withdrawal symptoms (2 or more together)"
11	Phy	Physical health problems from drinking
12	Emo	Emotional/psychological problems from drinking

Note: categorical values are assigned as – 1 for No and 1 for Yes In case of ALDX, -1 for ≤ 3 and 1 otherwise

**Table 2:** Twelve genome datasets with phenotype variables

No.	Label name of phenotypic traits	Proportion of classes			Total records
			
		No. of "-1"	No. of "-1"	"-1" vs. "1"	
1	Deceased	1202	2	601.0	1204
2	ALDX	273	927	0.3	1200
3	Binge	711	312	2.3	1023
4	Blackouts	624	399	1.6	1023
5	Morning	627	395	1.6	1022
6	Craving	826	197	4.2	1023
7	Pers	495	528	0.9	1023
8	Narrow	766	218	3.5	984
9	GUATD	767	256	3.0	1023
10	WDSX	776	235	3.3	1011
11	Phy	833	190	4.4	1023
12	Emo	746	277	2.7	1023
